# The Strasbourg Visual Scale: A Novel Method to Assess Visual Hallucinations

**DOI:** 10.3389/fpsyt.2021.685018

**Published:** 2021-06-09

**Authors:** Anne Giersch, Thomas Huard, Sohee Park, Cherise Rosen

**Affiliations:** ^1^University of Strasbourg, INSERM U1114, Strasbourg, France; ^2^Department of Psychiatry, University Hospital of Strasbourg, Strasbourg, France; ^3^Department of Psychology, Vanderbilt University, Nashville, TN, United States; ^4^Department of Psychiatry, University of Illinois at Chicago, Chicago, IL, United States

**Keywords:** hallucinations, psychosis, visual perception, scale, phenomenology, imagery, verbal report

## Abstract

The experience of oneself in the world is based on sensory afferences, enabling us to reach a first-perspective perception of our environment and to differentiate oneself from the world. Visual hallucinations may arise from a difficulty in differentiating one's own mental imagery from externally-induced perceptions. To specify the relationship between hallucinations and the disorders of the self, we need to understand the mechanisms of hallucinations. However, visual hallucinations are often under reported in individuals with psychosis, who sometimes appear to experience difficulties describing them. We developed the “Strasbourg Visual Scale (SVS),” a novel computerized tool that allows us to explore and capture the subjective experience of visual hallucinations by circumventing the difficulties associated with verbal descriptions. This scale reconstructs the hallucinated image of the participants by presenting distinct physical properties of visual information, step-by-step to help them communicate their internal experience. The strategy that underlies the SVS is to present a sequence of images to the participants whose choice at each step provides a feedback toward re-creating the internal image held by them. The SVS displays simple images on a computer screen that provide choices for the participants. Each step focuses on one physical property of an image, and the successive choices made by the participants help them to progressively build an image close to his/her hallucination, similar to the tools commonly used to generate facial composites. The SVS was constructed based on our knowledge of the visual pathways leading to an integrated perception of our environment. We discuss the rationale for the successive steps of the scale, and to which extent it could complement existing scales.

## Foreword

“The observer of the vision—like the psychotic with his visual hallucinations—knows that he cannot walk around the vision to view it from the back. A vision and a visual hallucination have no back; they are transparent to the observer, in the sense that there is nothing to discover about the vision that is not already immediately “visible.” A vision does not occupy space in the visible world in the same way that other things do. A vision rarely blocks the view of other things; in fact, other things don't even relate to a vision spatially in terms of “next to,” “in front of,” or above (1).”

## Introduction

Visual hallucinations (VHs) are perceptions that arise independently of an external stimulation. VHs are considered to be transdiagnosic, heterogeneous, multimodal sensory and perceptual experiences that often occur concurrently with auditory, tactile, olfactory or gustatory alterations (2, 3). VHs can range from simple fleeting shadows or various shapes to static cardboard-like images or even dynamic interactive scenes that vary in regards to specificity of detail. A number of studies have emphasized the fact that visual hallucinations are more frequent in individuals with schizophrenia than one might think (4–9). They appear to be underestimated and it has already been argued that we may require additional tools to avoid missing them (10). For example in 1989, Bracha et al. (5) showed that in individuals with chronic schizophrenia, 43% of the visual hallucination cases were first documented during research rather than during usual clinical investigations, suggesting that individuals with psychosis do not report their visual hallucinations spontaneously, and that psychiatrists may not ask systematically. Until recently, visual hallucinations had been mainly explored in degenerative pathologies like Parkinson, dementias, especially the Lewy Body dementia, or eye diseases (9), and in the context of hallucinogenic compounds such as LSD (11) and it is a common belief that visual hallucinations is a marker of a neurological rather than psychiatric disease (6). Yet, in schizophrenia, even if visual hallucinations are less frequent than auditory hallucinations, they would be present in around 27% of the individuals (6). We initially built the scale for individuals with schizophrenia, but it should be noted that the scale can be used with any individual with visual hallucinations who has difficulty to describe them.

It is surprising that individuals with schizophrenia do not report visual hallucinations more spontaneously, and less so than auditory hallucinations. It is all the more surprising that the mechanisms proposed to explain the emergence of hallucinations, even though diverse, are usually proposed to be similar for visual or auditory hallucinations (12–21). The main difference between visual and auditory hallucinations is observed for complex visual hallucinations, i.e., with nameable and meaningful forms. Complex, nameable hallucinations appear to be a marker of neurodevelopmental perturbations (22), more so than unformed hallucinations, or auditory hallucinations. It might explain why visual hallucinations are often associated with a worse outcome than auditory hallucinations (7, 8, 23). However, the aim of the new scale we propose here is not to help describing hallucinations that can be easily verbalized, but rather to support the communication of those hallucinations, or the properties of the visual hallucinations, that are difficult to describe. (e.g., irregular shapes, contours, hues, or movements that do not correspond directly to verbal or conceptual categories). The present paper is neither aimed at discussing the mechanistic origin of the hallucinations, but to review the rationale that leads us to propose a new approach to help individuals describing their hallucinations. It should be noted from the onset that this new approach is not aimed at replacing existing scales but at complementing them. It is meant to help individuals to describe their visual hallucinations when they have difficulties to describe them verbally.

When Waters et al. (6) reviewed the literature in 2014, they concluded that visual hallucinations in schizophrenia usually have the quality of real perceptions, detailed and solid, anchored in the environment. Fully-formed hallucinations are more commonly described than unformed hallucinations. Yet, one might wonder if visual hallucinations that are unformed and difficult to describe are more often missed than visual hallucinations that can easily be labeled. Early research with mescaline-induced hallucinations found that visual hallucinations were more prevalent, that these experiences were difficult to verbally describe, and that drawing and pictures provided a description where words could not (24). Recently van Ommen et al. (25) described a wide spectrum of visual hallucinations in individuals with psychosis. As reported in (6), complex hallucinations were the most frequent ones. However, they were often accompanied by simple, geometrical hallucinations. An important point was that those simple hallucinations were rarely the only hallucinations experienced by individuals, which might be another reason why they are not reported: it might be the case that individuals prioritize the description of easy-to-verbalize hallucinations, at the cost of the others. In addition, although the study confirmed that hallucinations were perceived as being as real as real perceptions, they often lacked details. The sample of individuals in the van Onmen's study was relatively small, but there is several reasons to believe that a number of unusual hallucinations may be missed in clinical practice. For example, Coughlan et al. (26) asked young people how they understand or “make sense” of their unusual perceptual experiences. Unusual experiences are not necessarily hallucinations, they can be simple distortions in perception. Yet the way the participants in this study interpret their perception illustrates the difficulties encountered when trying to capture the experience of the participants. About two thirds of the participants in Coughlan et al.'s study were confused or unsure about how to make sense of their experience, and sometimes attributed their experience to their imagination. This may contribute to the known loss of insight of the participants, which might lead to difficulties distinguishing between usual and unusual experiences in some patients (27). On the clinician's side it is also difficult to interpret the individuals' descriptions, like the following, reported by Freedman & Chapman in 1973 (28): ≪ Maybe I'm not very sensitive to sight... I have been short of a little blurry... I keep thinking maybe I'm tired... the other night in front of the television, I felt a sort of blurring like that. ≫ As a matter of fact there seems to be a close link between mental imagery and hallucinations (29–31), which makes it difficult for both the clinician and the patient to distinguish between the two. Imagery is associated with the activation of the same visual areas as real perception (32, 33), and it is no surprise that subjects with enhanced mental imagery tend to develop more visual hallucinations (29–31).

VH exist along a continuum, with phenomenological similarities of spontaneous mental imagery, dreams, hypnagogic-hypnopompic, and hallucinogenic experiences (34, 35). Persons who experience VH are more likely to remember words as pictures and experience difficulty distinguishing visual mental images and perception, suggesting a propensity to visually process information (14). VHs also involve a loss of agency and volitional control that is typically coupled with an emotionally charged reaction. A recent review (36) explored points of convergence and divergence in the phenomenological gestalt and processing of hallucinations, mental imagery and dreams and concluded that when distilled, the core phenomenological characteristics are similar. The salient evanescent and sometimes bizarre qualities of these phenomena that are considered “subcategories of a larger category” may also share similar mechanistic brain activities associated with consciousness, which will be discussed in more detail in a following section (37, 38).

The close link between hallucinations and disorders of the sense of self in individuals with schizophrenia may be yet another reason for the individuals' difficulty to describe their visual hallucinations. There are multiple hypotheses regarding the definitions and entanglements of self-disorder and psychosis. Early phenomenological psychopathologists at the Heidelberg School conceptualized self-disturbances, the Ichstörungen model, as psychotic symptoms (39, 40), whereas, the Ipseity model conceptualizes self-disorder as an independent and distinct phenomena that can be present within the psychotic experience but is considered a non-psychotic phenomena (41, 42). Self-disturbance is an anomalous experience that involves alterations in one's sense of self, others and the environment in which sense of ownership of thoughts and sensory experiences are no longer perceived within the context of mineness and dissolution of ego-boundaries. Attenuated ego boundaries as sub-clinical phenomenological changes in the basic symptom structure are often experienced throughout the course and include prodrome, acute symptom exacerbation and residual states (43–45). Self-disturbance, although present in psychosis in general, is more prevalent within the schizophrenia spectrum and may serve as a phenotypic marker of vulnerability for specific symptoms and behavioral features (46, 47). Self-disturbances and visual/multimodal hallucinations involve a deliquesce in the “perception-in-action” framework resulting in the underlying loss of embodied perspective (48).

Self-disturbances are closely associated with visual perceptual anomalies, even though this relationship may not seem obvious at first sight. Visual perception is experienced from our own first-person perspective, and there is usually no ambiguity regarding the frontier between ourselves and the external world. This appears to be different in individuals with schizophrenia. As described above, individuals with schizophrenia experience instability in their bodily self experience (49–54), inability to distinguish one's own self-boundary from the external world (55) and have a difficulty to take a first-person perspective (56–58). It has been proposed that these difficulties lead to hallucinations (54, 59, 60). Conversely, it can be argued that individuals with psychosis may experience difficulties defining their hallucinations when the border between the outer and inner world is blurred (55). A blurred border between the inner and outer world is likely to happen in acute phases, i.e., when hallucinations are the most prevalent. This leads to another problem, which is that often individuals with psychosis are asked to report their hallucinations after the acute stage when their symptoms have stabilized, i.e., when their state has changed, and possibly their perception of themselves and the outer world. It may be difficult for those individuals with strong disorders of the self to define their hallucinations clearly and to report them, and even more so at a distance from the acute episodes. It is probable that those hallucinations that are clearly experienced as coming from the outer world are those that are more easily reported (6). These myriads of difficulties that prevent detailed reporting and definition of visual hallucinations motivated us to build a new scale, especially as those visual hallucinations might be closely related to self disorders.

Another motivation at the root of the new scale is the complexity of the visual system, its consequences for our understanding of how visual perception becomes conscious, and the known impairments of visual processing in schizophrenia. Visual processing is our main sensory access to the world and more than 50% of the brain is devoted to the processing of visual information (61). Even though we have the impression of having a direct access to the world, visual information is often ambiguous, only partly visible. Many processing steps are required for visual information to be disambiguated. For example it has been shown that the detailed processing of visual information can require up to 300–500 ms (62). Another example is the huge impact of perceptual ambiguity on EEG signals (63, 64). Those examples illustrate the constraints put on the visual system when we try to make sense of our environment. It is thus no surprise that visual processing entails several parallel specialized pathways and many processing steps. Thereafter we summarize those steps.

Visual information is thought to be processed by specialized neurons with small receptive fields in the primary visual cortex (65). Those neurons code for selective information like orientation, color, luminance, spatial frequency. Information is then processed in the ventral and dorsal pathways (66). The ventral pathway conveys information toward the anterior part of the temporal lobe. Local contour information must be grouped together to access objects' forms, while being segregated correctly from other objects and from the background (67). Once the form of the objects has been accessed to, it is compared with representations of forms in memory. It is only when a close fit has been found that the object can be named (68). Importantly, color and form are processed in separate pathways (69, 70) and also require integration mechanisms for objects to be perceived as a whole. An organization in parallel pathways is the rule in the visual system, and is observed also in the visual dorsal pathway, which deals with spatial information, representation of objects for action, and possibly 3D representations of objects (71–74). In all, having access to the objects in the environment requires the integration of information across multiple pathways. This modular organization has a number of implications for how hallucinations can develop. First, it means we do not have direct and instant access to integrated objects, i.e., with all their attributes of surface, form and motion. Moreover, a number of authors suggested that it might be possible to become conscious of only partially integrated objects (75–77).

The questions raised about how we perceive objects in healthy volunteers are even more justified in the case of individuals with schizophrenia. Schizophrenia is a disorder affecting the sense of self and higher-order cognitive functions, and perception is often believed to be preserved. However, there is a number of studies showing that this is not the case, to the point that it is impossible to review all aspects here. We can only refer the reader to existing reviews (78–84). In 2016, Silverstein (84) has proposed a unified model of visual perturbations in schizophrenia. Interestingly he suggests a parallel with paintings of George Inness, which can help us to have a glimpse into how the world may look like for an individual experiencing VH: dimmed luminance, blurred contours, ambiguous forms, lack of spatial coherence. On top of these impairments of static visual processing, individuals with psychosis also experience impairments in dynamic environments related to eye movements disturbances (85–88), motion processing difficulties (89), and processing in time in general (90, 91). All these visual impairments, whether in space or in time, are usually related to clinical disorganization (78, 90), and this may explain why these two domains of research, visual perception and hallucinations, have remained separated. As a matter of fact, it does not seem that visual impairments are directly responsible for visual hallucinations [but see (92, 93)], which likely involves a difficulty to decouple imagery and perception. However, perception perturbations may still have an impact on the experience of visual hallucinations and the ability of individuals to report them. If individuals with psychosis have difficulties to perceive the outer world in a clear and organized fashion, there is a high probability that their visual hallucinations are similarly fuzzy or noisy. Once again, this might bias reports toward those hallucinations that are easily verbalized, whereas those that are less nameable would be missed. The latter would fit with clinical practice that suggests that individuals with schizophrenia sometimes have a hard time when trying to describe what they see. Additionally, little is known about the detailed visual distinctions and demarcation at the seam between the VH and the environment.

Visual impairments may make it difficult for individuals with schizophrenia to describe their hallucinations. In addition, verbalization may also have an impact *per se*. Individuals with schizophrenia show broad communication difficulties (94, 95) but more specifically, impaired access to the lexicon and word-finding difficulties in these individuals pose unique challenges for describing their experiences (96). These problems are further compounded by cognitive impairments including working memory and attention in this population (97–99). Describing internal sensations that do not fit neatly into available conceptual categories is difficult for most people. For example, most people have difficulty communicating the taste of a new wine or the olfactory landscape of an unfamiliar place. Nor do we find it easy to describe interoceptive sensations or for that matter, emotions. We simply do not have words for all the sensations in the world. However, it is possible to match internal representation of a person to an external representation through a matching-to-sample paradigm. Instead of asking people to provide verbal labels (especially when there are not any), one can present a visual stimulus for the participants to endorse or reject on the basis of their own internal sensations. Such strategies can be employed to reconstruct internal, subjective experiences.

In all, it is this background literature that leads us to propose a new kind of visual hallucinations scale, the Strasbourg Visual Scale, that relies on the step-by-step presentation of distinct physical properties of visual information, to help individuals describe their visual hallucinations.

## Methods

We are aware that proposing real pictures to the individuals with psychosis may be misleading or might bias the individual when choosing a description. The aim of the scale is not to create an exact replica of the hallucinations but to approximately match the participants' visual experiences of the physical characteristics of the hallucinations. The participants are made aware of these limitations beforehand. The scale is built by offering a maximum of four choices at each step, to make it as simple as possible. Each choice is illustrated by a picture which clarifies what type of physical characteristic is to be defined by the participants. Given the difficulties of the participants to describe what they see, this strategy is chosen to minimize the number of choices and reduce possible hesitations. For the same reason we offer simple choices rather than letting the participants e.g., draw their hallucinations. Importantly each choice of feature is progressively integrated within the picture proposed to the participants, meaning that the hallucination is composed step by step, as with a facial-composite approach.

**Figure 1:**
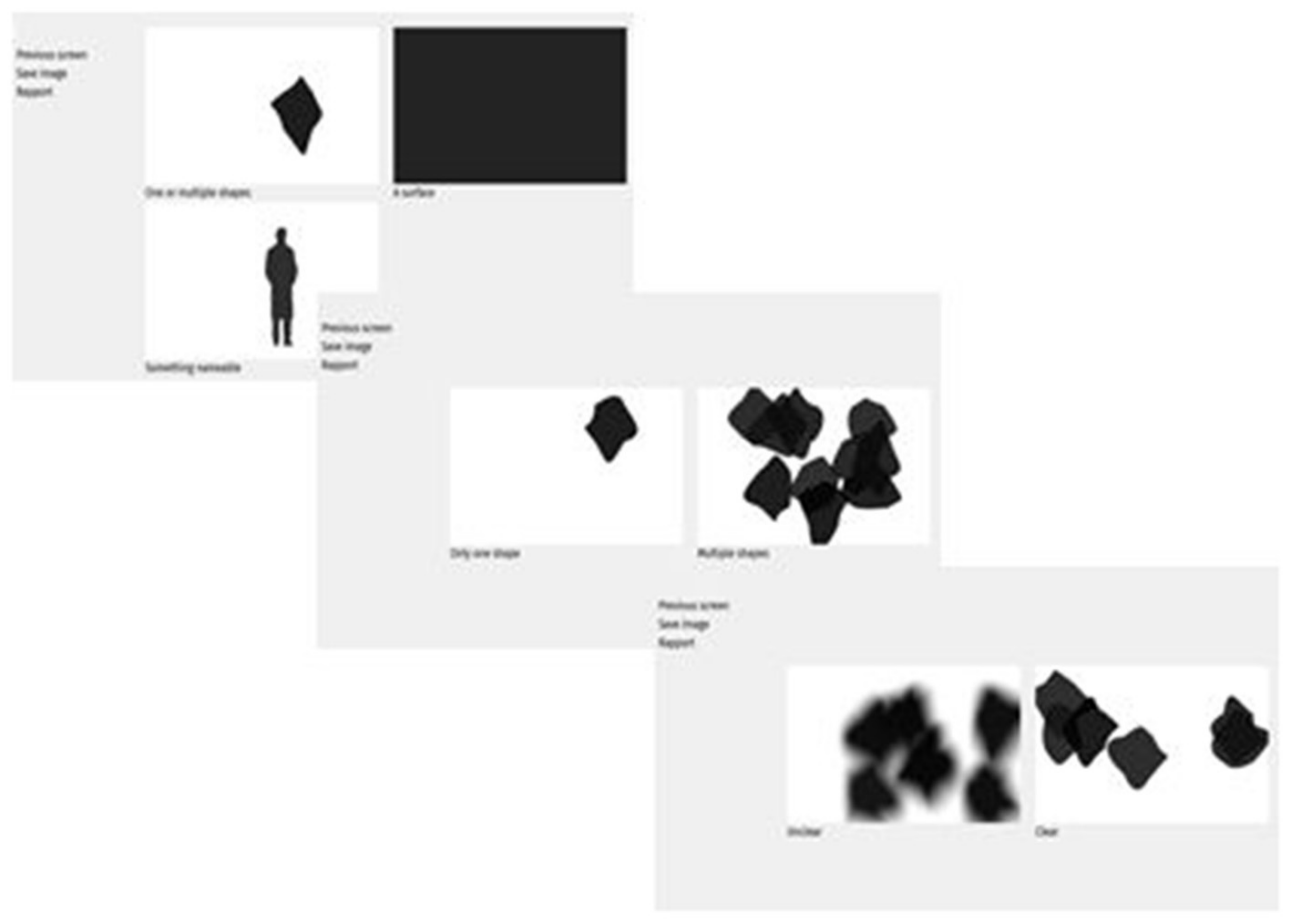
Illustration of three successive choices aimed at describing a shape.

The steps in the scale are modeled on the specialized visual processing pathways. We focus mainly on the ventral pathway, i.e., the processing of objects form, because spatial information (processed in the dorsal pathway) is less relevant to the identity, shape, texture of the VH. In addition, the spatial location of the VH may be more optimally investigated in different environments, e.g., virtual reality. First we take into account the separation between form and surface, and ask patients to choose to describe either a shape or a surface. We also ask whether the form can be nameable or not, so that its physical characteristics can be defined. Subsequent steps depend on the choices of the patients. An example of successive choices for a shape is illustrated Figure 1.

Thereafter we describe the sequence of questions and choices posed to the subjects. The duration of the test depends on the participants' choices, i.e., whether he or she wishes to describe only one form, or e.g., several forms and a surface, but in all the test duration is between 3 and 10 min at the most. The short duration of the test, the fact that there is only a limited number of choices on each screen, and the illustrations, make the test easy to run for both the participants and experimenters or caretakers, who do not need a special training. The main explanations about the aims of the test and how it works are integrated at the beginning of the scale, and theoretically the participants can self-administer the scale. However, caretakers are advised to accompany participants, in order to collect any additional information that could be triggered by running the scale. It is thus preferable that the experimenters or caretakers already have some experience in the clinical investigations of hallucinations. It should be noted that we do not ask questions about the content of the hallucinations, because we consider that this information can be better captured by existing scales. The present tool is aimed at providing information that is difficult to capture by verbal interviews. The test has been developed with the second author, who has created all the pictures, and a license has been filed (registered on November 8, 2019, at the French Software Protection Agency, under the reference number FR.001.460017.000.S.P.2019.000.10000). The software will be made available at no cost to anyone ready to sign a software license agreement.

**Figure 2:**
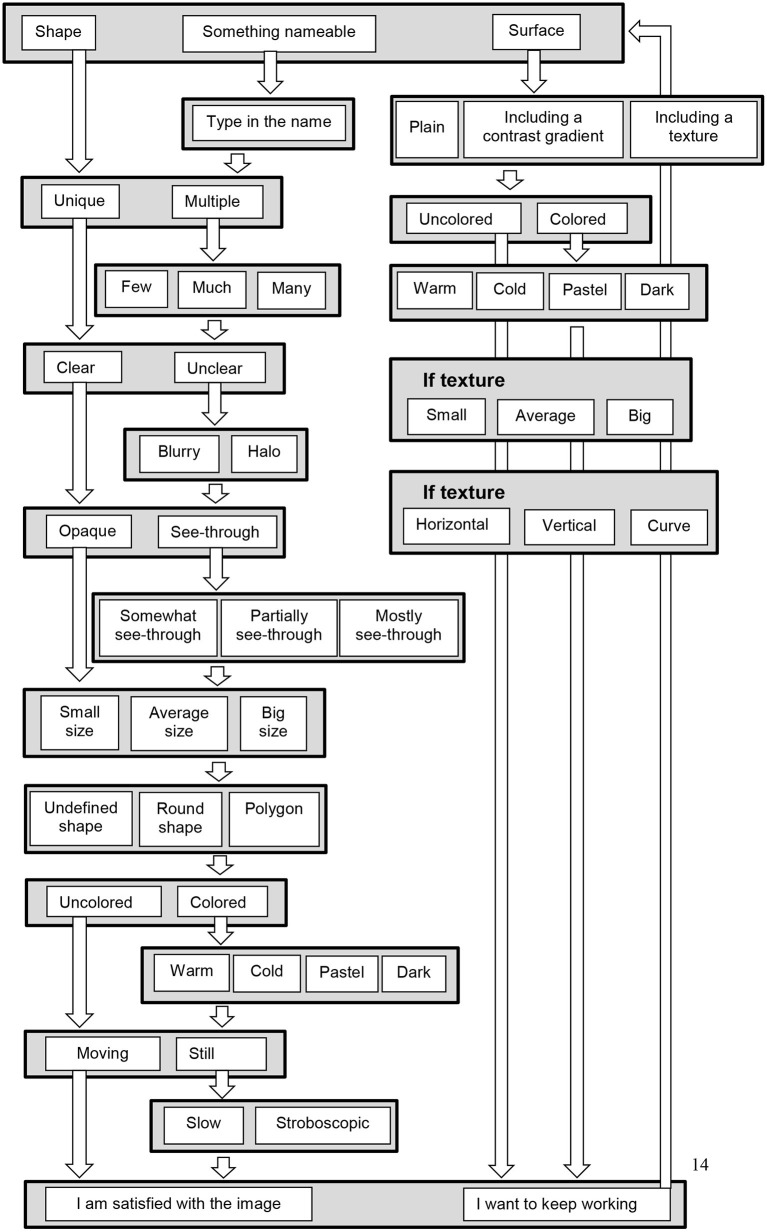
Flowchart questions in the SVS, when the first choice is a shape, something nameable, or a surface. At each stage choices are made by clicking on one of the displayed possibilities, as shown in Figures 1 and 3. Some of the questions are asked only when a preceding response justifies the question: for example if the participant chooses “colored,” then and only then a question on color types is displayed (warm, cold, pastel, and dark color). Once a picture is completed, it is possible to add shapes or a surface. Some additional (verbal) questions are asked at the end of the scale, as detailed in the text.

**Figure 3:**
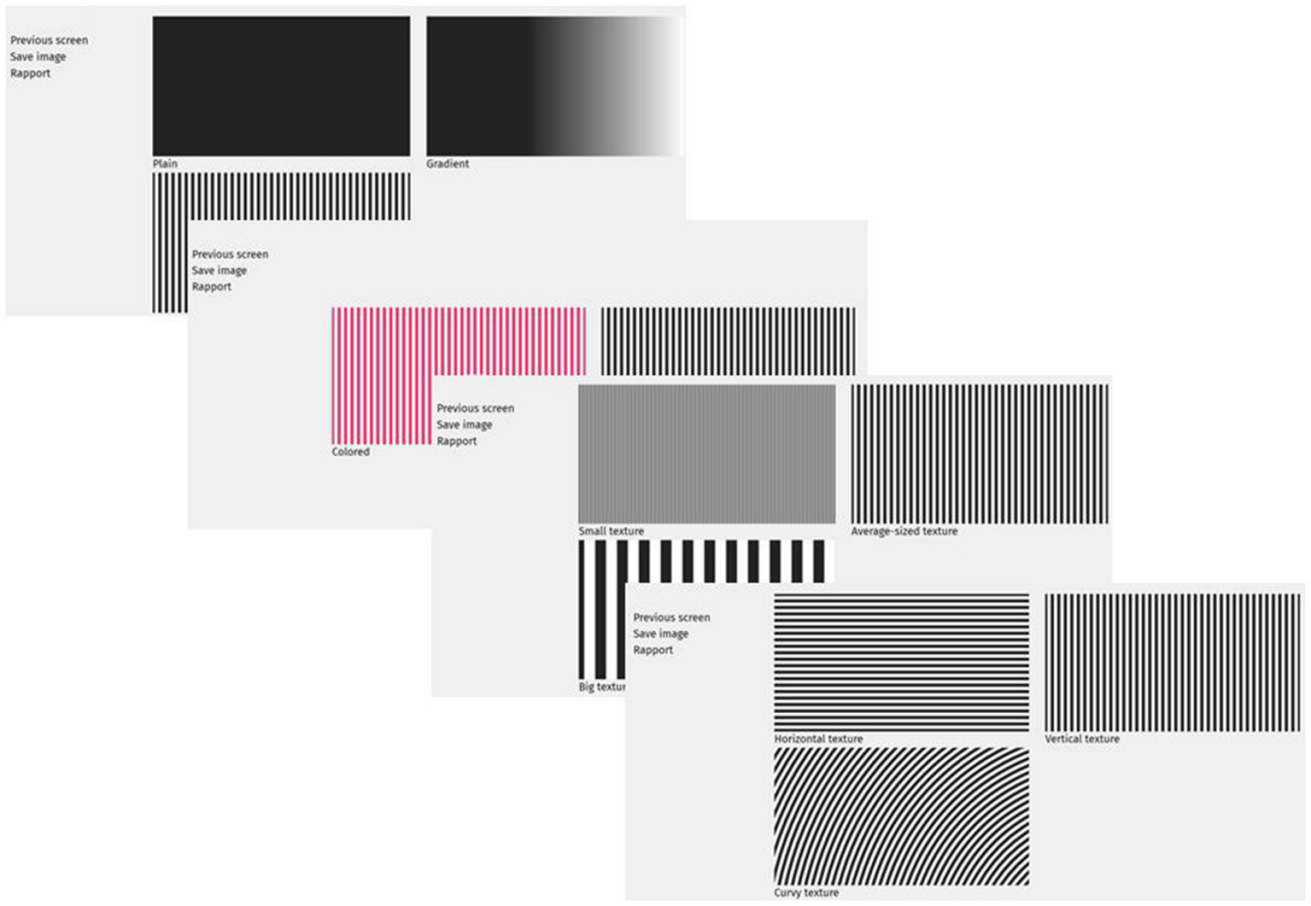
Illustration of four successive choices aimed at describing a surface.

For a shape, the participants indicate successively (1) whether the shape is unique or whether there are multiple shapes, (2) whether the shapes are clear or unclear, (3) whether they are opaque or see-through, (5) their size (small, average, big), (5) whether the shapes are undefined, round, or polygons, (6) colored or uncolored, (7) moving or still (Figure 2). Each of these successive steps consists in one screen with as much illustrations as there is choices, i.e., between 2 and 3 for the described choices. In the whole scale, there is never more than 4 illustrations at the same time on the screen. The participants make their choice by clicking on the picture corresponding to his or her choice. This then triggers the next screen with the following choice. There is additional questions as a function of some of the choices on the preceding questions. The exhaustive list of the illustrated choices are illustrated in Figure 2. An example of successive choices is illustrated in Figure 3.

The participants can superpose different shapes and surfaces if they wish, by going back to the beginning of the scale. Our scale is a prototype, and additional questions on the spatial position of the visual hallucination, and the possible integration with auditory hallucinations, are asked verbally, as in typical scales: the participants are asked to indicate (1) whether the created picture occupies the whole visual field, a large or a small part of the visual field, (2) where the picture is (in the middle, on the left or right side, top or bottom part of the visual field), (3) in the head or outside the head. The participants can specify whether they also hear voices (4) inside or outside the head, (5) whether the image and the voice comes from the same place, (6) if the voice is heard long before or a little before the image, (7) whether the intensity of the voice impacts the image. Our prototype can and should be expanded by integrating 3D information, space and sound. What we generally propose here is to complement verbal descriptions with techniques that stand out from verbal interviews when trying to capture the experience of the participants.

## Expected Results

**Figure 4:**
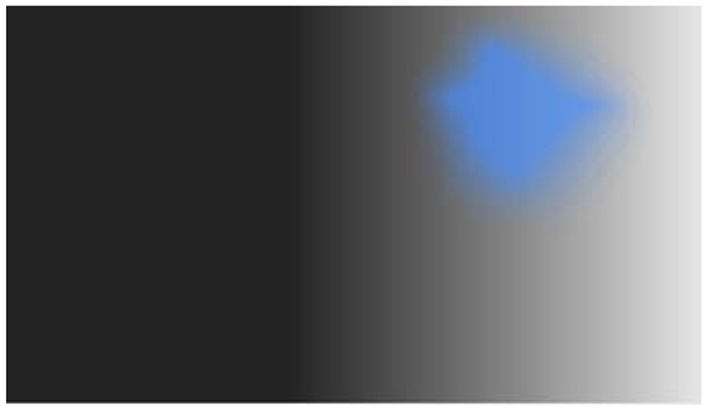
Example of one outcome of the visual hallucination scale (the example is fictitious, but similar to real outcomes obtained during clinical interviews).

One possible example of hallucination visualization is illustrated in Figure 4. The figure integrates the successive choices of the participants during the scale. The scale has now to be validated in a large group of individuals with experiences of VH. As already emphasized, a limit of the method is that choices are suggested explicitly instead of the participants freely describing his or her hallucinations. However, the results can indicate which pathways are involved in the hallucinations (form or not, color or not, and motion or not). The results can then be used to make hypotheses about the source of the hallucinations in the brain. This can in turn be confirmed or disconfirmed with imaging techniques, to orient research and therapeutic approaches.

## Discussion and Conclusion

In conclusion, we propose a novel approach to help those individuals who have difficulty describing hallucinations or when there are no appropriate verbal categories to describe hallucinated images. As noted at the beginning of the manuscript, our aim is not to replace existing scales. It remains critical to question the patient about the content, intrusiveness, or attributions of his or her perceptions. Our approach is specifically targeting hallucinations that do not map onto words or VHs that are difficult to describe. A first way to verify the usefulness of the scale will be to examine to which extent the scale helps to collect information about the hallucinations that were not obtained verbally. If this is the case, it may provide a more complete picture of the experience of the participants, by combining the results of the scale with other collected information about the hallucinations.

Defining the physical characteristics of the perceptions should help us to identify their source in the visual cortex and may contribute to decipher the emergence of hallucinations by complementing imaging approaches. A moving surface without shape would orient toward the dorsal pathway, whereas a static, colored shape would orient toward the ventral pathway. Such simple distinctions can be verified with imaging methods. The scale may also be used to define the impact of visual perception impairments. Combinations of ill-defined shapes and surfaces that appear successively instead of simultaneously would indicate that the hallucinated images are poorly integrated in space and time. In turn such results would indicate an influence of perception disorders on how hallucinations are perceived: a deficit in visual integration may contribute to the hallucination. As emphasized above hallucinations are supposed to arise as a result of a confusion between imagination and perception (14), and may be related to prior expectations and excessive top-down effects (100). However, such explanations may not apply to undefined or poorly integrated shapes: it is unclear why patients should expect or imagine an undefined form. Hallucinations of poorly defined shapes may rely on distinct or additional mechanisms, from those originating clear and nameable hallucinations.

What exactly happens for such unclear hallucinations is unclear, and the SVS scale may help. The difficulty stems from unsolved questions regarding the relationship between visual integration and consciousness. Given the need to integrate a large number of physical properties in order to identify objects, and given the fact that these properties are processed massively in parallel, it is unclear what happens at the subjective, conscious level if these visual mechanisms are affected at the same time as top-down, attention mechanisms. Fahrenfort et al. (101) have used multivariate classification analyses of electroencephalogram to show that the integration of surface and contour occurred even when consciouness access was disrupted. They suggested that what becomes conscious is only a part of what is processed in the brain [see also (102)]. Hence, whether information has to be integrated before becoming (at least partly) conscious, whether consciousness is an all-or-non-phenomenon, and whether top-down attention is really required for consciouness to emerge, is all a matter of debate. These questions may impact how hallucinations develop. Specifying which type of simple form is perceived with the SVS scale and combining it with visual perception investigations may help to explore how hallucinations relate with visual perception impairments. The scale may thus both contribute to solve questions about the relationship between consciousness and perception mechanisms, and to identify specific mechanisms of hallucinations in some patients.

It remains to be seen if helping individuals with VH to communicate their experience will help them to better manage the experience, by making it a defined object rather than a strange, scary impression that cannot be described in words.

## Data Availability Statement

The original contributions presented in the study are included in the article/supplementary material, further inquiries can be directed to the corresponding author/s.

## Author Contributions

SP, CR, and AG discussed the initial idea during an ICHR meeting. AG developed the idea and built the architecture of the scale with TH, who designed the pictures used in the scale and programmed the scale. CR helped to refine the steps of the scale. AG used the scale as an addition to her clinical evaluation of individuals with hallucinations, and wrote the first draft of the manuscript. CR and SP both provided substantial additions to the initial version. All authors contributed to the article and approved the submitted version.

## Conflict of Interest

The authors declare that the research was conducted in the absence of any commercial or financial relationships that could be construed as a potential conflict of interest.
